# Cryobiopsy: Should This Be Used in Place of Endobronchial Forceps Biopsies?

**DOI:** 10.1155/2013/730574

**Published:** 2013-08-27

**Authors:** Edmundo R. Rubio, Susanti R. le, Ralph E. Whatley, Michael B. Boyd

**Affiliations:** Section of Pulmonary, Critical Care, Environmental and Sleep Medicine, Virginia Tech Carilion School of Medicine—Carilion Clinic, Roanoke, VA 24014, USA

## Abstract

Forceps biopsies of airway lesions have variable yields. The yield increases when combining techniques in order to collect more material. With the use of cryotherapy probes (cryobiopsy) larger specimens can be obtained, resulting in an increase in the diagnostic yield. However, the utility and safety of cryobiopsy with all types of lesions, including flat mucosal lesions, is not established. *Aims*. Demonstrate the utility/safety of cryobiopsy versus forceps biopsy to sample exophytic and flat airway lesions. *Settings and Design*. Teaching hospital-based retrospective analysis. *Methods*. Retrospective analysis of patients undergoing cryobiopsies (singly or combined with forceps biopsies) from August 2008 through August 2010. *Statistical Analysis*. Wilcoxon signed-rank test. *Results*. The comparative analysis of 22 patients with cryobiopsy and forceps biopsy of the same lesion showed the mean volumes of material obtained with cryobiopsy were significantly larger (0.696 cm^3^ versus 0.0373 cm^3^, *P* = 0.0014). Of 31 cryobiopsies performed, one had minor bleeding. Cryopbiopsy allowed sampling of exophytic and flat lesions that were located centrally or distally. Cryobiopsies were shown to be safe, free of artifact, and provided a diagnostic yield of 96.77%. *Conclusions*. Cryobiopsy allows safe sampling of exophytic and flat airway lesions, with larger specimens, excellent tissue preservation and high diagnostic accuracy.

## 1. Introduction

The traditional approach to the diagnosis of visible endobronchial disease has been through forceps biopsy (FB) via a flexible bronchoscope with a diagnostic yield of 65% [1]. There is evidence that the size of the biopsies correlates with the diagnostic yield: larger samples obtained through a rigid bronchoscope have a yield of 78% [1]. Previous studies have concluded that the diagnostic yield of bronchoscopy is augmented when multiple procedures are performed, including brushings, needle aspirates, and washings [2]. However, performing all of these increases the operative time, overall cost, and potentially the risk of anesthesia-associated complications. 

The use of the cryotherapy probe allows sampling of endobronchial tumors, producing well-preserved tissue, which tends to be of superior quality to that obtained by flexible FB. Recent studies demonstrate that cryobiopsy (CB) samples are devoid of the crush artifact commonly seen in biopsies obtained with traditional forceps [3, 4]. CB also yields much larger samples than FB. The cryotherapy probe has not been routinely used as a biopsy instrument primarily due to concerns that the immediate pulling of a frozen probe could lead to significant complications, such as bleeding and airway tears. These concerns persist despite the demonstrated safety of the cryoprobe in providing for recannalization of obstructive airway tumors [3, 4]. 

The putative advantages of performing CB of endobronchial lesions are improved diagnostic yields, reduced operative time, and potentially decreased complications. Additionally, the cryotherapy probe may facilitate the sampling of airway lesions positioned tangentially to the bronchoscope. Such lesions, especially if flat, are difficult to sample with the endobronchial forceps. 

Though there is a paucity of data on the subject, a recent study in Europe suggests that the use of cryotherapy probes to sample endobronchial exophytic visible lesions is safe and yields well preserved tissue samples [4]. Additionally, another recent subsequent larger multicenter trial of CB demonstrated a superiority of CB over traditional forceps biopsy [5]. While this study was well designed, it was limited only to the diagnosis of malignancies, and patients did not get submitted to both procedures, being randomized to CB or FB. Furthermore, the number of biopsies obtained was left to the discretion of the operator, though limited to no more than four. Finally, the time of freezing was not standardized, though they report that in general around 2-3 seconds were enough. Notably, the longer the freezing time is the larger the tissue sample one could expect. However, tissue characteristics and applied pressure may also influence the sample size. Unfortunately, an analysis of how the number of biopsies or freezing time may have impacted the diagnostic yield is not provided in detail, other than a general statement that the average number of biopsies was 3.24 ± 1.16 versus 3.45 ± 0.95 for CB and FB, respectively. While one might conclude that CB should be the preferred method based on this study, they also reported a significant higher rate of bleeding complications in the CB group. Moreover, one could argue that the diagnostic advantage of CB could be eliminated by increasing the number of FB samples obtained, with yet a lesser overall bleeding risk. Cryobiopsies have been routinely and safely performed at our institution, both in exophytic as well as nonexophytic airway lesions and for benign and malignant diseases. We have also standardized the technique and have been securing the diagnosis in most of our patients by utilizing multiple sampling techniques in the same patient. To assess the potential benefits of CB to sample these lesions, as opposed to traditional FB, a retrospective review was conducted to evaluate the safety of this procedure, volumes of material obtained, the types of lesions amenable to biopsy in this fashion, and the overall diagnostic yield. 

## 2. Subjects and Methods

An institutional retrospective analysis was performed to all patients that underwent bronchoscopic CB from August of 2008 until August of 2010, either singly or in combination with FB. The study was approved by the “Carilion Clinic Institutional Review Board,” approval number N/A with approval filed by name (safety and benefits of endobronchial cryobiopsy). Only patients (inpatients and outpatients) that received care by a newly established interventional pulmonary service were included, regardless of lesion location, appearance (exophytic versus flat lesions), and diagnoses, including both benign and malignant disease. Both flexible and rigid bronchoscopic procedures were included. All biopsy procedures were performed either directly or supervised by three pulmonologists, two of which were specifically trained and experienced in interventional pulmonology. Both FB and CB were performed in many cases as part of the training of staff and pulmonary fellows in both techniques. The decision to perform CB was at the discretion of the interventional pulmonologist.

### 2.1. Bronchoscopy

*Flexible bronchoscopies* were performed using either an Olympus Diagnostic Video Bronchoscope with an outside diameter of 4.9 mm or 5.1 mm and a working channel of 2.0 mm or an Olympus Therapeutic Video Bronchoscope with an outside diameter of 6.0 mm or 6.3 mm and inner working channels of 3.0 mm and 3.2 mm, respectively. All flexible bronchoscopies were performed in the endoscopy suite with the use of laryngeal mask airways and an “anesthesia driven” protocol with deeper sedation but avoiding full paralysis. *Rigid bronchoscopies* were performed when patients were anticipated to require additional interventions for an airway obstructing lesion. In these cases, the patients were intubated using a Bryan-Dumon series II rigid bronchoscope (Bryan Corporation), with the CB being directed through the Olympus Therapeutic Flexible Video Bronchoscopes. When rigid bronchoscopy was performed, the procedure was carried out in the operating room, under general anesthesia.

### 2.2. Biopsy Procedures

CB was performed using the ERBOKRYO CA system (ERBE USA Incorporated Surgical Systems). Two probes were utilized, one of 2.4 mm in diameter and one of a smaller 1.9 mm diameter, the latter allowing its use through the smaller diagnostic bronchoscope. Endobronchial FB was obtained using a Boston Scientific Radial Jaw 3, 2.0 mm single use needleless forceps (Boston Scientific Corporation). 

Endobronchial CB is performed in our institution with the following protocol; the cryotherapy probe is placed in direct contact with the lesion that is being sampled, and when approaching exophytic lesions, the probe is frozen for 3 seconds (or until the initiation of the frosting at the tip of the probe is visible). With flat lesions a freezing time of 2 seconds is used (to minimize freezing and remove normal mucosa adjacent to the lesion), and at times the freezing time is extended to 3 seconds based on the observation of the operator. After this short period of freezing, the bronchoscope is removed en bloc with the cryotherapy probe. The sample obtained is placed in 0.9% sodium chloride solution through passive thawing and then placed in 10% formalin. FBs were performed by passing the forceps into the bronchoscope working channel or via the rigid bronchoscope barrel, opening the forceps, advancing the bronchoscope or forceps onto the lesion, closing the forceps thus grasping, gently tugging as to tear the tissue while retrieving the forceps through the working channel or barrel, and then placing in 10% formalin. In the patients that were studied, a minimum of 3 endobronchial forceps biopsies were performed initially, and subsequently 2 cryobiopsies were obtained in each case. As noted, in all cases the number of FB exceeded the number of CB. 

### 2.3. Tissue Preparation and Pathology

 All samples were fixed in 10% formalin for at least 24 hours. Pathological analysis was routinely performed by staff pathologists. Using centimeters (cm), biopsies were measured in three dimensions (length, width, and depth) prior to sectioning for microscopic evaluation. When tissues were limited and volumetric measurements could not be reasonably performed (a total of only 3 cases), specimen slides were retrospectively reviewed by a pathologist, who measured the length and width of material on each slide. Each dimensional measurement was divided by the total number of slides for each specimen to establish an average length (example: FB length on slide 1 was 0.02 cm; FB length on slide 2 was 0.01. Therefore, the average FB length of this specimen was 0.02 + 0.01/2 = 0.015 cm). Tissue depth on the slides was standardized to 0.01 cm. Pathological interpretation was reported in the patients' electronic medical record.

### 2.4. Final Diagnosis

All results were classified as malignant or nonmalignant based on histology. A diagnosis of malignancy was confirmed with the clinical findings. A nonmalignant diagnosis was established by close clinical and radiological followup to monitor stability. This followup extended to a mean duration of 22.9 months (range, 14 to 36 months) until the submission of data.

### 2.5. Safety

Charts were reviewed looking for potential complications to include bleeding, bronchospasm, or respiratory distress. Bleeding severity was defined as minimal if <30 cc; moderate if between 30–100 cc; or severe if >100 cc. The presence of bronchospasm was defined by postprocedure wheezing and/or the administration of bronchodilators prior to discharge from the recovery area. Respiratory distress was defined as requiring additional supplemental oxygen due to hypoxemia, increase work of breathing, and admission to hospital or, if already an inpatient, transfer to a higher level of care.

### 2.6. Statistics

 In patients where both CB and FB were performed, the Wilcoxon signed-rank test was utilized to analyze the volumes of biopsies obtained by each method.

## 3. Results

**Figure 1:**
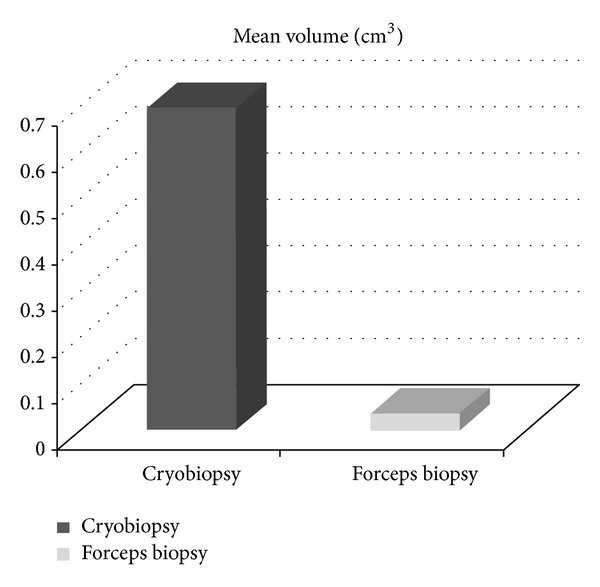
Comparison of cryobiopsy versus forceps biopsy (*n* = 22 patients).

Over a two-year period, 166 bronchoscopies were performed at our institution by the interventional pulmonary service. With three pulmonologists routinely performing CB, 31 patients were identified as undergoing CB either singly or in combination with FB. Patient characteristics (sampling technique utilized, pathological diagnosis, location of lesion, volume of material obtained, complications, and type of lesion—exophytic or flat) are summarized in Tables 1 and 2. Twenty-two patients underwent both biopsy techniques (Table 1). The mean volume of material obtained by CB in these patients was significantly larger (0.696 cm^3^) when compared with the FB (0.0373 cm^3^), with a *P* value = 0.0014 (Figure 1). The nine remaining cases only had CB (Table 2). The comparative analysis of the data of the group with both procedures demonstrated a diagnostic yield of 100% for CB, while FB failed to adequately diagnose only one patient (with a large exophytic lesion) resulting in diagnostic yield of 95.45%. The diagnostic yield for all patients undergoing CB was 96.77%. A diagnosis was not obtained in only one case in which necrotic debris was identified. All samples obtained by CB demonstrated excellent tissue preservation, and were deprived of any noticeable artifact. Only one patient demonstrated minor bleeding as a complication of CB. Finally, while CB was confirmed to be safe and effective in sampling exophytic lesions (18/31), similar findings were demonstrated in biopsying flat irregular mucosal lesions (13/31). In this regard, one patient was diagnosed with an in situ carcinoma. This patient had a right upper lobe posterior segment narrowing without any evident exophytic lesion or clear mucosal abnormality other than mild bleeding emanating from this subsegment, indicating the need to biopsy this area. The CB probe facilitated easier sampling of this area with no associated complications.

## 4. Discussion

### 4.1. Technique of Cryobiopsy

CB can be performed using both the flexible and rigid bronchoscope. The use of the cryotherapy flexible probe guided by a flexible bronchoscope allowed endobronchial cryobiopsies of central airways, as well as more distal airways, provided that the lesion is visible on bronchoscopic evaluation. Though all lesions were deemed approachable at the outset, one operator noted that it was difficult to flex the bronchoscope in order to biopsy a lesion in the right upper lobe even with the use of the smaller diameter cryotherapy probe (although this was eventually accomplished). Thus, one potential limitation is that lesions located in airways requiring significant flexure of the bronchoscope may not be reached with the current cryotherapy probes. Operators found that sampling lesions lying parallel to the axis of the bronchoscope (rigid and flexible) was much easier with the cryotherapy probe than with the endobronchial forceps. 

A disadvantage of CB via a flexible bronchoscope is that the frozen probe with the attached biopsy material cannot be removed through the working channel. In order to remove the specimen from the airway, it is necessary to remove both the bronchoscope with the cryoprobe and the attached specimen en block. The procedure is repeated by reinserting the bronchoscope and the cryoprobe. Endotracheal intubation could be utilized to minimize the possibility of vocal cord and airway trauma. In this regard, Hetzel and colleagues in their recent study of CB actually indicated that a disadvantage of CB was that it is recommended to intubate all patients, either with a flexible endotracheal tube or through rigid bronchoscopy [5]. While we agree with them in that the complication risk from intubation is small, we do not consider it negligible. Additionally, such practice is certainly not commonly accepted in many institutions. In our experience, the use of a supraglottic airway mask to perform routine bronchoscopies can many times be advantageous, as is the case when performing bronchoscopies with CB. It provides similar advantages to endotracheal intubation yet carries a lower risk of direct damage to the vocal cords or trachea.

### 4.2. Diagnostic Yield and Tissue Volume

Our data demonstrates that the diagnostic yield and accuracy of CB is comparable and possibly superior to traditional endobronchial FB. This has also been suggested by our colleagues in Europe. The diagnostic yield of FB is generally accepted to be below 80%, though it can improve with the performance of additional sampling techniques [1, 2]. CB has a previously reported sensitivity of 89%, when biopsying exophytic malignant lesions, and more recently an overall 95% diagnostic yield for all malignant lesions [3, 5]. Our diagnostic yield is comparable or higher than those previously reported for both CB and FB. For FB one reason may be related to sampling technique and the use of deeper sedation, allowing more accurate targeting of the suspicious lesion. Repeated biopsy sampling of each lesion may have resulted in a greater volume of material, which has been demonstrated to improve the diagnostic yield [6]. Interestingly, Hetzel et al. also demonstrated a higher yield of FB when this was performed under rigid bronchoscopy (reported at 89.5%) and noticed that CB did not provide a statistically significant advantage in these cases. The authors comment also on the fact that “due to general anesthesia and a reduced breathing amplitude, positioning of the forceps becomes easier, thus diminishing the benefit of cryobiopsy” [5]. One must also wonder if this simply led to more FB samples. In their study the number of biopsies was left to the bronchoscopist discretion, and the number of biopsies obtained or an analysis of this during the rigid bronchoscopy cases is not provided [5]. 

While others have examined the importance of biopsy tissue area, we analyzed volume [2, 4, 6]. Larger specimens (hence, larger volumes) have shown to increase diagnostic yields [1]. Although our study is unable to determine the influence of biopsy depth on diagnosis, it is necessary to consider that FB may provide insufficient depth to obtain malignant cells invading the submucosa. Unfortunately, Hetzel et al. did not evaluate biopsy sample sizes; hence their increased diagnostic yields with CB cannot conclusively be correlated to larger samples [5]. We demonstrate that the volume of tissue obtained with CB is significantly larger than that of endobronchial forceps biopsies. It is important to note that one patient had near total removal of an exophytic airway lesion on the first CB. This resulted in a high volume of biopsy material (12.25 cm^3^). A statistical analysis using only the remaining 21 patients undergoing both CB and FB still demonstrated mean volumetric superiority with CB (0.146 cm^3^versus 0.0375 cm^3^, *P* = 0.0018). While the amount of tissue obtained on biopsy may improve the diagnostic yield, it also has therapeutic implications, especially in those with malignancy. As cancer therapy becomes more individualized, larger tissue samples may prove useful in studies such as mutational analysis and genetic profiling. Finally, it was noted that there was good concordance of diagnosis between the CB and the FB, with the exception of one case, namely, the one that was almost completely removed with the CB. It is rather unusual to see that with such short freezing times an endobronchial tumor would almost completely detach from its base. This suggests that this tumor was very friable and possibly with advanced necrosis. The latter may explain why a more superficial biopsy, such as expected with a forceps, would fail to demonstrate adequate characteristics to affirm a diagnosis of malignancy, having in this case only demonstrated atypical cells. 

### 4.3. Safety

Biopsies performed with the cryotherapy probe proved extremely safe. One minor complication was reported, related to a small bleed that resolved spontaneously, following instillation of cold saline. This patient was on clopidogrel bisulfate therapy but presumably had stopped this a week earlier. While in general we do not perform biopsies in patients on this therapy, the pressing need to start treatment on some of these patients may indicate the need to proceed with the biopsy even while actively taking such drug. Additionally, one could argue that the vasoconstrictive properties of the cryobiopsy may ameliorate excessive bleeding. Of interest in this regard, it must be noted that the study by Hetzel et al. included 5 patients on Clopidogrel Bisulfate, but they do not report specifically on any associated bleeding in these patients. Additionally, they report a comparable rate of severe bleeding of 17.8% and 18.2% for FB and CB, respectively, although the overall bleeding rate was significantly higher with CB [5]. In those cases performed under rigid bronchoscopy, one could argue that the higher bleeding risk and lack of significant increase in diagnostic yield with CB would indicate that under such circumstances CB would not be justifiable. Their findings regarding bleeding complications are in contrast to ours, noticing that we had no significant bleeding complications. While our study may be limited by its smaller size, one alternative explanation could be that their cases may have been subject to more sampling or perhaps longer freezing times. Finally, there were also no mucosal tears complicating any of our CB, including flat mucosal lesions. Airway bleeding and mucosal tears may have been avoided due to brief freezing times (no longer than 3 seconds) limiting the depth and extension of tissue adherence to the cryoprobe. Even though our limited freezing time may have led to obtaining smaller tissue samples, the volumes obtained were both adequate for diagnosis and larger than the traditional forceps biopsies. No patients experienced bronchospasm following CB, and recovery times were similar to traditional bronchoscopy. A final consideration, albeit outside of the scope of this study, is the fact that an “anesthesia driven” protocol (with deeper sedation than in a bronchoscopist led conscious sedation protocol) to facilitate our cases allowed for a safe and controlled environment, such that all subjects, even if with advanced lung disease, were able to tolerate the procedure well and return to their prior baseline immediately following their procedure and as noted with no associated complications. While some may remain concerned with the use of deeper sedation and general anesthesia (as in the case of our rigid bronchoscopy cases), Hetzel et al. study also showed that over 45% of their patients underwent general anesthesia with also reportedly no associated complications [5]. 

### 4.4. Cost and Procedural Time

It is necessary to point out that the CB will be enduring over time. If multiple sampling techniques could be avoided, it is foreseeable that the operative time would be decreased when utilizing CB, as this single technique would achieve adequate tissue samples to secure a diagnosis. Additionally, the fact that the cryotherapy probe is reusable may also translate in cost savings (the same two flexible cryoprobes were used in all the cases, with no breakages during any of the procedures). An additional consideration is that if CB is able to provide improved yields, perhaps this can also be accomplished with a single sample versus two or more. Considering this to be true, then procedure times and cost could be minimized, though given the fact that the same probe is being used and time needed for each additional CB is short (estimated at less than 1 minute) the potential benefits would likely be negligible. In any case, our study was not designed to evaluate this question, as all patients had 2 CB which were submitted in a single preparation tube and analyzed in toto. It is nevertheless suggested that the operator, as she/he becomes more experienced, could accurately make the decision to obtain a simple specimen if that is visually of large enough volume and does not appear clearly necrotic. It is our hope that in future studies, we will be able to better address some of these considerations.

## 5. Conclusions

While our study has limitations due to the small numbers, it adds to a slowly growing body of the literature supporting the use of cryobiopsy as an important tool in the diagnosis of endobronchial disease. Additionally, our study provides further insight in how to safely perform the procedure in order to limit complications. We also demonstrate that this technique is useful to sample both exophytic, as well as visible flat irregular mucosal lesions. This technique is safe and appears to provide equivalent or superior diagnostic yields when sampling malignant lesions, as suggested by others, with our study also highlighting its utility to diagnose benign airway lesions. Additionally, as cancer targeted therapies evolve, tissue biopsy volume may be crucial to obtain adequate DNA quantities for proper genotyping and patient management, and as noted we have demonstrated a significant advantage for CB in this regard. 

Finally, our study demonstrates that this procedure can be safely performed without the need to intubate patients, by using an anesthesia driven protocol with the placement of a supraglottic airway mask. 

We encourage further evaluation of this technique and hope that our study will help promote awareness of this emerging approach to endobronchial biopsies. We hope to continue to study the many advantages that CB has to offer, by increasing the number of patients in the studies. We would also welcome the development of smaller diameter probes, which may overcome any potential limitations of this approach to sampling lesions within upper lobe segments. 

## Figures and Tables

**Table 1:** Patient diagnosis (*n* = 22) according to biopsy type, location of lesion, biopsy material volume (cm^3^) corresponding to each biopsy modality, and type of lesion (exophytic or flat).

Cryobiopsy diagnosis	Forceps diagnosis	Location of lesion	Vol. (cm^3^) cryobiopsy	Vol. (cm^3^) forceps	Type of lesion
Squamous cell carcinoma	Squamous cell carcinoma	BI	0.4	0.2	Exophytic
Benign inflammation	Benign inflammation	RUL	0.06	0.018	Flat
Necroinflammation	Necroinflammation	RUL	0.048	0.001	Flat
Necroinflammation	Necroinflammation	Carina	0.018	0.002	Flat
Papillary squamous cell carcinoma	Papillary squamous cell carcinoma	RMS	0.702	0.004	Exophytic
Adenocarcinoma	Adenocarcinoma	RMS	0.02	0.00025	Exophytic
Adenosquamous	Adenosquamous	RLL	0.004	0.004	Flat
Squamous cell carcinoma	Squamous cell carcinoma	RUL	0.032	0.144	Exophytic
Small cell	Small cell	LMS	0.027	0.021	Exophytic
Benign	Benign	RMS	0.018	0.009	Flat
Squamous cell carcinoma	Squamous cell carcinoma	LLL	0.112	0.006	Exophytic
Poorly differentiated NSCLC	Poorly differentiated NSCLC	LLL	0.024	0.04	Exophytic
Small cell	Small cell	RUL	0.03	0.016	Exophytic
Squamous cell carcinoma	Squamous cell carcinoma	RMS	0.96	0.009	Exophytic
Adenocarcinoma	Adenocarcinoma	LLL	0.045	0.032	Flat
Carcinoid	Carcinoid	RLL	0.39	0.234	Exophytic
Adenocarcinoma	Adenocarcinoma	RLL	0.024	0.006	Flat
Benign	Benign	LUL	0.05	0.032	Flat
Squamous cell carcinoma	Squamous cell carcinoma	RMS	0.042	0.006	Flat
Squamous cell carcinoma	Squamous cell carcinoma	RUL	0.04	0.001	Flat
Squamous cell carcinoma	Squamous cell carcinoma	LMS	0.016	0.003	Flat
Adenocarcinoma	Atypical	RMS	12.25	0.032	Exophytic

NSCLC: Nonsmall cell lung cancer; BI: bronchus intermedius; RUL: right upper lobe; RLL: right lower lobe; LUL: left upper lobe; LLL: left lower lobe; RMS: right main stem; LMS: left main stem.

**Table 2:** Patient diagnosis (*n* = 9) in patients who only had CB and no FB, location of lesion, biopsy material volume (cm^3^), and type of lesion (exophytic or flat).

Cryobiopsy diagnosis	Location of lesion	Vol. (cm^3^) cryobiopsy	Type of lesion
In situ squamous cell carcinoma	RUL	0.39	Flat
Acid fast bacilli	LMS	0.147	Exophytic
Necrotic debris	RLL	0.36	Exophytic
Small cell*	RUL	0.027	Exophytic
Small cell	LMS	2.0	Exophytic
Small cell	LLL	0.45	Exophytic
Squamous papillomata	Trachea	0.12	Exophytic
Adenocarcinoma	Carina	0.21	Flat
Small cell	LLL	0.168	Exophytic

RUL: right upper lobe; RLL: right lower lobe; LLL: left lower lobe; LMS: left main stem.

*Mild bleeding noted only in this patient.
